# Alpha-2-adrenergic agonists reduce resting energy expenditure in humans during external cooling

**DOI:** 10.1080/23328940.2024.2339781

**Published:** 2024-04-21

**Authors:** Clifton W. Callaway, Katharyn L. Flickinger, Alexandra Weissman, Francis X. Guyette, Ryann DeMaio, Andrea Jonsson, Victor Wu, Jenna L. Monteleone, Peter Prescott, Jonathan Birabaharan, Daniel J. Buysse, Philip E. Empey, Thomas D. Nolin, Raymond E. West

**Affiliations:** a Department of Emergency Medicine, University of Pittsburgh School of Medicine, Pittsburgh, PA, USA; b Center for Clinical Pharmaceutical Sciences, Department of Pharmacy and Therapeutics, University of Pittsburgh School of Pharmacy, Pittsburgh, PA, USA; c Department of Psychiatry, University of Pittsburgh School of Medicine, Pittsburgh, PA, USA

**Keywords:** Hypothermia, energy expenditure, dexmedetomidine, tizanidine, alpha-2-adrenergic drugs

## Abstract

Intravenous alpha-2-adrenergic receptor agonists reduce energy expenditure and lower the temperature when shivering begins in humans, allowing a decrease in core body temperature. Because there are few data about similar effects from oral drugs, we tested whether single oral doses of the sedative dexmedetomidine (1 µg/kg sublingual or 4 µg/kg swallowed) or the muscle relaxant tizanidine (8 mg or 16 mg), combined with surface cooling, reduce energy expenditure and core body temperature in humans. A total of 26 healthy participants completed 41 one-day laboratory studies measuring core body temperature using an ingested telemetry capsule and measuring energy expenditure using indirect calorimetry for up to 6 hours after drug ingestion. Dexmedetomidine induced a median 13% – 19% peak reduction and tizanidine induced a median 15% – 22% peak reduction in energy expenditure relative to baseline. Core body temperature decreased a median of 0.5°C – 0.6°C and 0.5°C – 0.7°C respectively. Decreases in temperature occurred after peak reductions in energy expenditure. Energy expenditure increased with a decrease in core temperature in control participants but did not occur after 4 µg/kg dexmedetomidine or 16 mg tizanidine. Plasma levels of dexmedetomidine but not tizanidine were related to mean temperature change. Decreases in heart rate, blood pressure, respiratory rate, cardiac stroke volume index, and cardiac index were associated with the change in metabolic rate after higher drug doses. We conclude that both oral dexmedetomidine and oral tizanidine reduce energy expenditure and allow decrease in core temperature in humans.

## Graphical abstract

**Figure:**
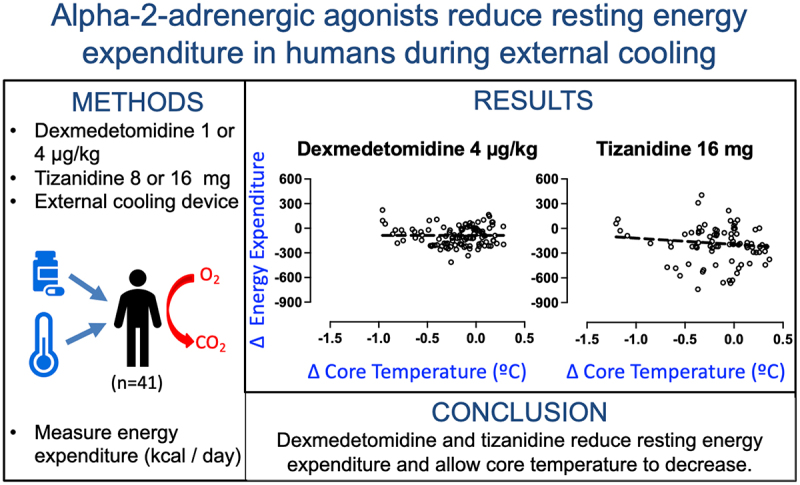

## Introduction

The metabolic rate of an animal can be measured by the interrelated rates at which it uses fuel (energy expenditure), utilizes oxygen, and excretes carbon dioxide. Reducing resting metabolic rate is an adaptation during environmental stress for many animals. Some animals exploit daily periods of low activity during which metabolic rate and temperature decrease below normal [1]. Hibernating animals create longer lasting reductions of metabolic rate and temperature. For example, bears during winter reduce their resting energy expenditure by 50–75% and their body temperature to 30–34°C for months [2]. Ground squirrels can reduce their oxygen consumption by more than 95%, associated with a reduction of body temperature to near freezing for days to weeks [3]. These strategies may improve survival in locations with large fluctuations in weather or food supply [4]. Under normal conditions, animals increase metabolic rate in response to cold in order to maintain core body temperature [5], and this increase is called cold-induced thermogenesis [6]. Cold-induced thermogenesis may include shivering, increased brown-fat metabolism, or other behaviors that vary between species. Hibernating animals may begin hibernation with a reduction in energy expenditure that is independent of temperature [2]. This temperature-independent reduction in resting energy expenditure is accompanied by inhibition of cold-induced thermogenesis, which allows core body temperature to decline [5,7]. Because metabolic rate also decreases at lower temperatures [5,8], declining body temperatures create a further temperature-dependent reduction in the metabolic rate of hibernating animals. The contribution of temperature-dependent and temperature-independent reductions of metabolic rate during torpor and hibernation varies between species [1,7].

Deliberately reducing metabolic rate in humans would prolong the duration of survival in resource-limited or extreme undertakings. For example, anesthesia and hypothermia are used to reduce metabolic rate in order to protect the brain and other organs during surgical procedures where blood flow needs to be interrupted for a short time [9]. Inducing a low metabolic rate state in non-anesthetized humans would make other applications possible. For example, astronauts might exploit drug-induced reduction in metabolism during long-duration space flight to conserve consumables, thereby reducing payload [10]. Similarly, reduced metabolic rate in the heart or brain when blood supply is reduced during a myocardial infarction or stroke could reduce organ damage while waiting for a reperfusion procedure [11,12]. Reduced oxygen utilization might also be beneficial in shock or lung injury [13]

Humans do not routinely exhibit hibernation, but artificial strategies including drugs can reduce metabolic rate. Humans also have robust cold-induced thermogenesis that increases metabolic rate when body temperature declines, with shivering being the most visible and energetic component. The temperature at which cold-induced thermogenesis is triggered can decrease in response to physiological states or after drugs [6]. Previously, we observed that titrated intravenous dexmedetomidine, an alpha-2-adrenergic receptor (A2AR) agonist, reduces oxygen utilization, induces sleep-like sedation, and facilitates induction of hypothermia by inhibiting shivering in healthy humans [14–17]. In order to develop a practical regimen for inducing a prolonged drug-induced metabolic reduction in humans, we tested whether drugs that could be self-administered by noninvasive routes also can reduce metabolic rate and inhibit cold-induced thermogenesis. We used indirect calorimetry to calculate energy expenditure as an index of metabolic rate. We hypothesized that A2AR agonists and surface cooling would reduce energy expenditure and core body temperature relative to baseline in healthy humans. This study measured the magnitude and duration of decreases in whole-body energy expenditure and temperature in healthy humans induced by single oral or sublingual doses of two A2AR agonists, the sedative dexmedetomidine and the muscle relaxant tizanidine, combined with surface cooling.

## Materials and methods

### Study design

The University of Pittsburgh Human Research Protection Office reviewed and approved the protocol. The study comprised five independent experiments. In each experiment, we assessed drug effect over time for each participant by comparing physiological measures collected for up to 6 hours after drug administration to baseline measures collected for 1 hour prior to drug administration. The baseline served as a within-participant control, and each participant had surface cooling device after drug administration. Each experiment tested a different drug or drug dose: (1) a control group who received no drug had physiological measures for 1 hour of baseline with no surface cooling followed by 4 hours with external surface cooling, (2) sublingual dexmedetomidine 1 µg/kg, (3) oral dexmedetomidine 4 µg/kg, (4) tizanidine 8 mg, and (5) tizanidine 16 mg.

### Study protocol

On arrival at the laboratory, participants ingested core temperature pills and the study team placed an intravenous catheter for blood draws. The study team placed all monitors. We recorded baseline metabolic rate and physiological measures for 60 minutes, after which the participants took the study medication. We recorded continuous metabolic rate and physiological measures for at least 4 hours after medication administration. Participants were allowed to eat and drink during the protocol. The study team assisted the participant to the toilet if needed during the protocol. Every 2 hours, participants were awakened if sleeping to answer questions and check on comfort. During these breaks, we removed the canopy.

A physician investigator allowed participants to leave when the participant felt that they were no longer experiencing any subjective effects of the drug and when their physiological measures were returning to normal. Thus, complete data collection was 4 hours for sublingual dexmedetomidine and control, and 5.5–6 hours for oral dexmedetomidine and tizanidine. The team removed the intravenous catheter and monitors. We confirmed well-being of participants by phone on the day after the protocol.

### Setting

We conducted experiments between 8 am and 6 pm in a laboratory with constant light (350–400 Lux) and ambient noise (55 dB). To minimize circadian variation between experiments, all participants received drugs between 10 am and 11 am. Participants were allowed to eat prior to arrival at the laboratory but were instructed to avoid caffeine the day of experiments. Ambient air temperature was mean 21.8 (SD 1.2) ºC and humidity varied from 26% to 63% (mean 51%, SD 10%). During study interventions, participants reclined on a stretcher with their head elevated to their comfort. Participants dressed in athletic clothes for all activities.

### Participants

We recruited healthy volunteers through advertisements posted in and around the University and via the University’s human research website (Pitt+Me). All volunteers provided written informed consent prior to screening for participation. Participants were required to be 18–55 years old, nonsmokers without sleep disorders or chronic health problems that would interfere with resting in the laboratory during a day-long protocol. We excluded volunteers who had allergies to study medications, who were taking medications that would interact with study medications, or who had claustrophobia that would prevent them from resting while being monitored.

On a screening visit, we confirmed that physical characteristics were in the target range: mass (>55 kg), body mass index (18.5 – 30 kg/m^2^), heart rate (50–100 beats/minute), and blood pressure (diastolic 60–90 mmHg, systolic 100–150 mmHg). We obtained a 12-lead electrocardiogram (ECG) and excluded volunteers with signs of atrioventricular conduction delay or resting dysrhythmias. Volunteers completed the Epworth Sleepiness Scale (ESS). We excluded volunteers who had excessive daytime sleepiness (ESS ≥ 11) [18]. To assess fitness, participants demonstrated their grip strength using a Jamar dynamometer (Chicago, IL). We measured maximum rate of oxygen consumption (VO_2_) by having volunteers complete a treadmill Bruce Protocol while breathing through a facemask into the Parvo TrueOne 2400 metabolic cart (Parvo Medics, Salt Lake City, UT). We excluded volunteers whose maximum VO_2_ was <1 SD below the mean or >2 SD above the mean for their age and sex [19], and also excluded volunteers whose maximum grip strength was not within 2 SD of the mean for their age and sex [20].

Volunteers who passed all of the screening evaluations were allowed to participate in the experimental protocol. Participants capable of becoming pregnant provided a negative urine HCG test on the day of participation. We allowed participants to test multiple drugs if visits were separated by a minimum of 2 weeks.

### Temperature measurement

To measure core temperature, participants swallowed a radiotelemetry thermometer capsule (HQI Inc., Palmetto, FL) or (eCelsius in research mode, BodyCap, Hèrouville Saint-Claire, France) that relayed deep gastric or enteral temperature to an external receiver every 15–20 seconds.

### Metabolic rate

Energy expenditure represents the amount of metabolic fuel consumed per unit time. We calculated the predicted resting energy expenditure (pREE) for each participant based on height, mass, age, and sex [21].

For females: pREE = 9.99 x mass + 6.25 x height – 4.92 x age – 161

For males: pREE = 9.99 x mass + 6.25 x height – 4.92 x age + 5

We measured O_2_ consumption (VO_2_) and CO_2_ excretion (VCO_2_) using the metabolic cart to provide indirect calorimetry estimates of total energy expenditure. Participants laid under a clear canopy during experiments. The canopy had one air inlet and one outlet through which room air was drawn at a constant rate (15–25 lpm) by a fan on the metabolic cart. Concentrations of O_2_ and CO_2_ in the sampled air and in room air, along with air flow rate, were measured continuously and means recorded every minute. We calibrated the metabolic cart at the beginning of each experiment with gas standards (AirGas USA, Plumsteadville, PA). VO_2_, VCO_2_, resting energy expenditure, and respiratory exchange ratio (RER) were calculated from these measures using the formulas of Weir and assuming a fixed contribution of protein to energy expenditure of 12.5% [22].

$$VO_{2}=\;\left({\left[O_{2}\right]}_{\mathrm{inspired}}-\;{\left[O_{2}\right]}_{\mathrm{expired}}\right)\;\;x\;\mathrm{flow}\;\mathrm{rate}$$

$$\mathrm{VC}O_{2}=\;\left({\left[CO_{2}\right]}_{\mathrm{inspired}}-\;{\left[CO_{2}\right]}_{\mathrm{expired}}\right)\;x\;\mathrm{flow}\;\mathrm{rate}$$

$$\;\begin{array}{rl} & \mathrm{Energy}\;\mathrm{Expenditure}\;\;=\;\left(3.941\;x\;VO_{2}\;+\;1.106\;x\;\mathrm{VC}O_{2}\right)\\ & \;\;\;\;\;\;\;\;\;\;\;\;\;\;\;\;\;\;\;\;\;\;\;\;\;\;\;\;\;\;\;\;\;\;\;\;\;\;\;\;\;\;\;\;\;\;\;\;\;\;\;\;/\left(1+0.082\;x\;0.125\right) \end{array}$$

$$\mathrm{RER}\;=\;\mathrm{VC}O_{2}/\;VO_{2}$$

### Physiological measures

We recorded continuous ECG rhythm using chest surface electrodes. A finger probe was used to record continuous pulse oximetry saturation (SpO_2_) and pulse plethysmography. We recorded respirations using a circumferential chest strap just below the nipple line. We calculated heart rate from each beat-to-beat interval on continuous ECG or plethysmography data. These continuous measures were converted to digital recordings at 1000 samples/second with an analog to digital converter (AD Instruments, Sydney, Australia).

We measured and recorded blood pressure every 15 minutes using an automated sphygmomanometer (Edan Instruments Inc, Shenzhen, PR China). We measured cardiac stroke volume and calculated cardiac output using the bioreactance method (Noninvasive Cardiac Output Monitor, NICOM, Cheetah Medical, Newton Center, MA), which has been described in detail and which has good correlation to themodilution or Fick methods in patients who are not in cardiogenic shock [23,24]. These data were expressed as stroke volume index (SVI) and cardiac index (CI).

### Shivering

Observers rated the Bedside Shivering Assessment Scale (BSAS) every 15 minutes. This scale ranges from 0 (no shivering) to 3 (uncontrolled shivering in all extremities) [25].

### Surface cooling

We placed a commercial gel-adhesive pad (Arctic Sun, Franklin Lakes, NJ) on the back of each subject. Each pad has a surface area of 0.17 m^2^, and we used one or two pads for each participant depending on the size of their torso (8%–15% of total body surface area), in order to cover the entire back. A control console circulated water through the pad so that water was in close proximity to skin. We adjusted temperature of the water during the experiment as low as possible as allowed by participant comfort. Initial water temperature was set to 15°C below torso skin temperature. We then decreased water temperature by 5°C every 10–20 minutes until the water temperature was 4°C or until the participant reported that the pad was too cold or had any signs of shivering. When participants reported that pads were too cool, we increased water temperature by 5°C every 10–20 minutes.

### Drug administration

We used the intravenous liquid formulation of dexmedetomidine (Par Pharmaceuticals, Chestnut Ridge, NY; Lots 30,609, 49329, and 55,121) and the tablet form for tizanidine (Apotex Corp, Weston FL, Lot TH1991). Because we administered these drugs by routes or doses that differ from their approved labeling, a physician was present during the experiments. Laboratory safety equipment included emergency life support drugs and equipment. Continuous respiratory and physiological monitoring were part of the protocol. A research team member escorted each participant whenever they stood lest they have orthostatic symptoms.

#### Sublingual dexmedetomidine

We dripped 1 mcg/kg dexmedetomidine (100 µg/ml) from a syringe onto the participant’s oral mucosa under the tongue. Participants were instructed to hold this liquid under the tongue until it was absorbed and not to swallow the liquid. All participants reported that they felt the liquid had disappeared within 2 minutes of administration.

#### Oral dexmedetomidine

We dripped 4 mcg/kg dexmedetomidine (100 µ/ml) from a syringe into the participant’s mouth. Participants were instructed to swallow this liquid followed by a rinse and swallow of water.

#### Oral tizanidine

Participants swallowed two or four 4 mg tablets of Tizanidine.

### Plasma drug levels

To determine plasma drug levels during the experiment, we drew 4 ml of blood into EDTA tubes from the intravenous catheter at various time points. For tizanidine and oral dexmedetomidine, we sampled blood at baseline, 30, 60, 90, 120, 180, 240 and 300 minutes. For sublingual dexmedetomidine, we expected absorption to be more rapid and therefore sampled blood at baseline, 15, 30, 45, 60, 90, 120, 180 and 240 minutes. We immediately centrifuged blood samples at 3000 × g for 20 minutes and aliquoted plasma into cryotubes. Plasma was stored at −40°C until assay.

Previously, we developed a LC-MS/MS assay and validated it according to the FDA Bio-analytical Method Validation Guidance for Industry for the quantification of dexmedetomidine in human plasma [26,27]. Briefly, 100 µL of plasma was protein precipitated using 500 µL of acetonitrile containing internal standard. The samples were centrifuged at 12,000 × g for 8 minutes. The supernatant was transferred and dried down at 38°C under nitrogen before being reconstituted in 75 µL of 95:5 (A:B, 0.15% formic acid: acetonitrile). Samples were then centrifuged again under the previously mentioned settings. Supernatant was then transferred to HPLC vials for analysis. A gradient elution was used to separate the analytes with a total runtime of 10 minutes. This assay showed excellent inter- and intra-day linearity, accuracy, and precision for dexmedetomidine.

In addition, the dexmedetomidine LC-MS/MS assay was used as a template and expanded for the quantification of tizanidine in human plasma. The sample preparation, LC parameters, columns, and gradient were the same for tizanidine and dexmedetomidine, however there were a few key differences. A LC-MS/MS system consisting of a Waters Acquity I-class UPLC and Thermo Scientific TSQ Quantis Plus that was equipped with a heated ESI (HESI) source was used for tizanidine, and the SRM transitions used for quantitation were m/z 254.1 → 44.0 for tizanidine and m/z 258.1→ 48.0 for d4-tizanidine. The concentration range for dexmedetomidine was 0.05–7.5 ng/mL while the concentration range for tizanidine was 0.2–100 ng/m. This assay showed excellent inter- and intra-day linearity (R^2^ = 0.9972), accuracy (>6.8%), and precision (>6.9%) [27].

### Data management

We collated the raw values for all recorded variables in 1-minute epochs. For measurements that were collected at higher sampling rates (e.g. heart rate), we recorded the arithmetic mean of values within the minute. We replaced artifactual data points and data points resulting from the monitor being disconnected with missing values using the following rules: Heart rate <30 or >120 (these values occurred when beat-to-beat interval was calculated from ECG artifact rather than R–R intervals); Respiratory rate <3 or >30 (these values occurred only when subjects were talking); Oxygen saturation <80% (this occurred only when blood pressure cuff inflated above the sensor or when the sensor was removed from the finger); Core temperature <35°C (this level of hypothermia never occurred during this study, and lower values reflected loss of telemetry signal); Metabolic measurements when FECO_2_ <0.5 (this low FECO_2_ occurs only when the canopy is removed from the participant). For analysis, we collected data into time epochs by averaging all of the baseline measurements into one epoch and averaging values after drug administration into sequential 30-minute epochs.

### Statistical analysis

#### Time-course of drug effect

For each experiment, we tested if drug affected energy expenditure and other variables, by analyzing changes in variables over time epochs after drug administration. We determined the main effect of time for metabolic rate, temperature, heart rate, systolic blood pressure and diastolic blood pressure using MANOVA. To determine which time epochs were different from baseline, we contrasted the mean and 95% confidence intervals for each variable within a time epoch with the mean for that variable during baseline. We graphed the mean and 95% confidence intervals of variables over time.

#### Comparisons between drugs

As a secondary analysis, we compared changes in variables between drugs and doses by calculating the maximum decrease from baseline and the mean deviation from baseline from 0 to 4 hours (area under the curve, AUC) for each physiological variable. We compared the maximal and the mean deviation in physiological variables between drug groups using one-way ANOVA. If there was a significant effect of drug group, we calculated pair-wise comparisons of means with Bonferroni correction for multiple comparisons to determine which drug group differed from the control group.

#### Association of energy expenditure with change in core temperature

In order to test whether there was evidence of cold-induced thermogenesis under each drug condition, we examined associations between change in energy expenditure with change in core temperature using ordinary least-squares regression. We also explored associations between temperature, energy expenditure, and other physiological variables.

#### Association of physiological changes with plasma drug concentrations

We examined associations between individual drug levels and changes in physiological variables using linear regression. Drug levels were expressed as AUC for plasma concentrations. We examined AUC for time intervals corresponding to the maximum duration of clinical sedation for each drug (0–2 hours for sublingual dexmedetomidine, 0–4 hours for 8 mg tizanidine, 0–5 hours for 16 mg tizanidine and 0–6 hours for oral dexmedetomidine) and also for a fixed time interval from 0 to 4 hours. Because results were similar for each comparison, we present only the latter. We compared this AUC to the mean deviation from baseline for each physiological variable for the same time interval.

We used complete data for analysis and did not impute or interpolate any missing values. Original sample size calculations selected 8 subjects per drug dose to have 90% power to detect a 18% change or larger in metabolic rate. We defined an alpha-error rate of 0.05 for significance. We performed all statistical analyzes using STATA SE 16.1 (College Station, TX), and prepared graphs using PRISM 9 (GraphPad Software, San Diego, CA).

## Results

We screened 32 volunteers, of whom 26 participated in 41 protocol days (19 participants tested one, 5 participants tested two, and 3 participants tested four drugs or doses). Characteristics of the participants are listed in Table 1. Groups were similar. We excluded six volunteers from participation: five whose maximum VO_2_ was >2 SD below the mean for age and sex and one whose ESS suggested excessive daytime sleepiness.

**Table 1.** Characteristics of participants.

Variable	Control	1 µg/kg Sublingual Dexmedetomidine	4 µg/kg Oral Dexmedetomidine	Tizanidine 8 mg	Tizanidine 16 mg
N	8	8	9	8	8
Age (years)	24 (3)	24 (4)	27 (10)	24 (3)	25 (4)
Female (%)	3 (38%)	2 (25%)	5 (56%)	1 (13%)	2 (25%)
Asian Black White Multiple/Other	3 1 4 0	1 0 6 1	0 3 5 1	4 1 2 1	1 2 4 1
Mass (kg)	78.8 (11.8)	75.4 (14.6)	72.5 (13.6)	77.7 (14.1)	74.6 (15.7)
Height (cm)	172 (9.2)	175 (8.6)	172 (8.0)	176 (8.2)	173 (8.8)
BSA (m^2^)	1.92 (0.18)	1.90 (0.21)	1.85 (0.20)	1.94 (0.21)	1.88 (0.22)
% Body Fat	20.1 (6.8)	16.1 (6.7)	18.8 (4.0)	14.4 (5.2)	16.2 (9.3)
VO_2_ max (ml/kg/min)	38.6 (4.9)	40.1 (12.0)	36.7 (3.6)	42.1 (6.8)	40.4 (6.5)
Predicted Basal Metabolic Rate (kcal/24 hours)	1688 (217)	1691 (230)	1582 (224)	1744 (206)	1606 (243)
Grip Strength (kg)	35.8 (7.0)	40.7 (11.7)	37 .0 (9.4)	39.1 (6.5)	33.9 (2.9)
Epworth Sleepiness Scale	5.5 (3 – 7)	7 (4.5 – 7)	3 (2 – 5)	3.5 (1.5 – 5.5)	3 (1.5 – 6)

Values are n (%), mean (SD) or median (IQR).

During baseline data collection, participants remained still with eyes closed. Energy expenditure and temperature changed little during this baseline. The measured energy expenditure during baseline for each study (Table 2) correlated well with the pREE (Table 1) (*r* = 0.709, *p* < 0.001).

**Table 2.** Maximum and mean drug-induced change in metabolic rate and temperature.

	Baseline Value	Lowest Value	Maximum Deviation (Lowest – Baseline)	%	Mean Deviation from Baseline (0–4 hours)	%
**Energy Expenditure (kcal/24 hours)**						
Control	1818 (1477 – 2184)	1493 (1308 – 1826)	−180 (−298 to −124)	−9%	−15 (−62 – 77)	−0.7%
Sublingual (1 µg/kg) Dexmedetomidine	1704 (1602 – 1864)	1320 (1275 to 1488)	−305 (−494 to −206)	−19%	−205 (−341 – 62)	−12%
Oral (4 µg/kg) Dexmedetomidine	1471 (1337 – 1695)	1238 (1220 – 1281)	−218 (−250 to – 118)	−13%	−108 (−138 to – 42)	−5%
Tizanidine 8 mg	1597 (1475 – 1794)	1434 (1229 – 1611)	−227 (−256 to −214)	−15%	−129 (−147 to −82)	−8%
Tizanidine 16 mg	1743 (1489 – 1984)	1239 (1194 – 1547)	−342 (−557 to −249)	−22%	−192 (−287 to −120)	−13%
**VO**_**2**_**(METS)**						
Control	0.88 (0.81 – 1.04)	0.79 (0.71 – 0.94)	−0.08 (−0.16 to −0.05)	−8%	0.00 (−0.03 to 0.04)	−0.5%
Sublingual (1 µg/kg) Dexmedetomidine	0.88 (0.81 – 1.00)	0.77 (0.61 – 0.82)	−0.18 (−0.22 to −0.11)	−18%	−0.10 (−0.16 to 0.05)	−11%
Oral (4 µg/kg) Dexmedetomidine	0.85 (0.81 – 0.97)	0.79 (0.75 – 0.82)	−0.11 (−0.13 to −0.06)	−12%	−0.05 (−0.08 to −0.02)	−5%
Tizanidine 8 mg	0.84 (0.82 – 0.96)	0.77 (0.70 – 0.83)	−0.13 (−0.14 to −0.11)	−14%	−0.07 (−0.08 to −0.04)	−8%
Tizanidine 16 mg	0.96 (0.90 – 1.01)	0.79 (0.70 – 0.92)	−0.18 (−0.21 to −0.09)	−19%	−0.09 (−0.12 to −0.02)	−9%
**Respiratory Exchange Ratio**						
Control	0.84 (0.81 – 0.87)	0.79 (0.75 – 0.81)	−0.06 (−0.07 to −0.05)	–	−0.01 (−0.04 to −0.01)	–
Sublingual (1 µg/kg) Dexmedetomidine	0.82 (0.80 – 0.86)	0.77 (0.61 – 0.82)	−0.07 (−0.09 to −0.04)	–	−0.03 (−0.05 to 0.01)	–
Oral (4 µg/kg) Dexmedetomidine	0.79 (0.76 – 0.82)	0.67 (0.64 – 0.82)	−0.05 (−0.06 to −0.05)	–	−0.02 (−0.04 to −0.02)	–
Tizanidine 8 mg	0.78 (0.77 – 0.79)	0.64 (0.62 – 0.69)	−0.03 (−0.07 to −0.03)	–	−0.01 (−0.03 to 0.01)	–
Tizanidine 16 mg	0.80 (0.74 – 0.86)	0.61 (0.57 – 0.71)	−0.06 (−0.08 to −0.05)	–	−0.03 (−0.05 to −0.01)	–
**Core Temperature (ºC)**						
Control	36.9 (26.6 – 36.9)	36.5 (36.3 – 36.9)	−0.0 (−0.4 – 0.00)	–	0.1 (0.1 to 0.2)	–
Sublingual (1 µg/kg) Dexmedetomidine	37.1 (36.8 – 37.2)	36.2 (36.0 – 36.8)	−0.6 (−1.0 to −0.4)	–	−0.2 (−0.5 to −0.1)*	–
Oral (4 µg/kg) Dexmedetomidine	36.7 (36.7 – 37.0)	36.4 (36.3 – 36.6)	−0.5 (−0.8 to −0.3)	–	−0.2 (−0.3 to 0.0)*	–
Tizanidine 8 mg	36.7 (36.6 – 36.9)	36.1 (35.9 – 36.2)	−0.7 (−1.0 to −0.4)	–	−0.0 (−0.1 to 0.1)	–
Tizanidine 16 mg	36.9 (36.7 – 37.1)	36.4 (36.2 – 36.8)	−0.5 (−0.6 to −0.2)	–	−0.2 (−0.3 to 0.1)	–

Per-subject median (IQR) values for baseline, minima and change in the 30-minute averages for each physiological variable. Maximum deviation is zero when the baseline value was the lowest recorded value. % Change is the median maximum absolute decrease in real variables. A symbol (*) indicates *p* < 0.05 for comparison of drug group relative to control.

### External cooling

We observed overt shivering (BSAS > 0) for 1 or 2 minutes on single occasions in 2 participants after 8 mg tizanidine, 2 participants after 16 mg tizanidine, 2 participants after sublingual dexmedetomidine and 1 participant after oral dexmedetomidine. Shivering immediately stopped when we raised cooling pad temperatures. Energy expenditure increased transiently during the minutes when shivering was recorded but was similar to the mean of the epoch during the minutes prior to and after shivering. During the active cooling, mean water temperature in cooling pads was for 11 (SD 7)ºC sublingual dexmedetomidine, 17 (SD 7)ºC for oral dexmedetomidine, 12 (SD 8)ºC for tizanidine 8 mg, 15 (SD 6)ºC for tizanidine 16 mg, and 15 (SD 2)ºC for control.

### Time-course of drug effect

**Figure 1.**
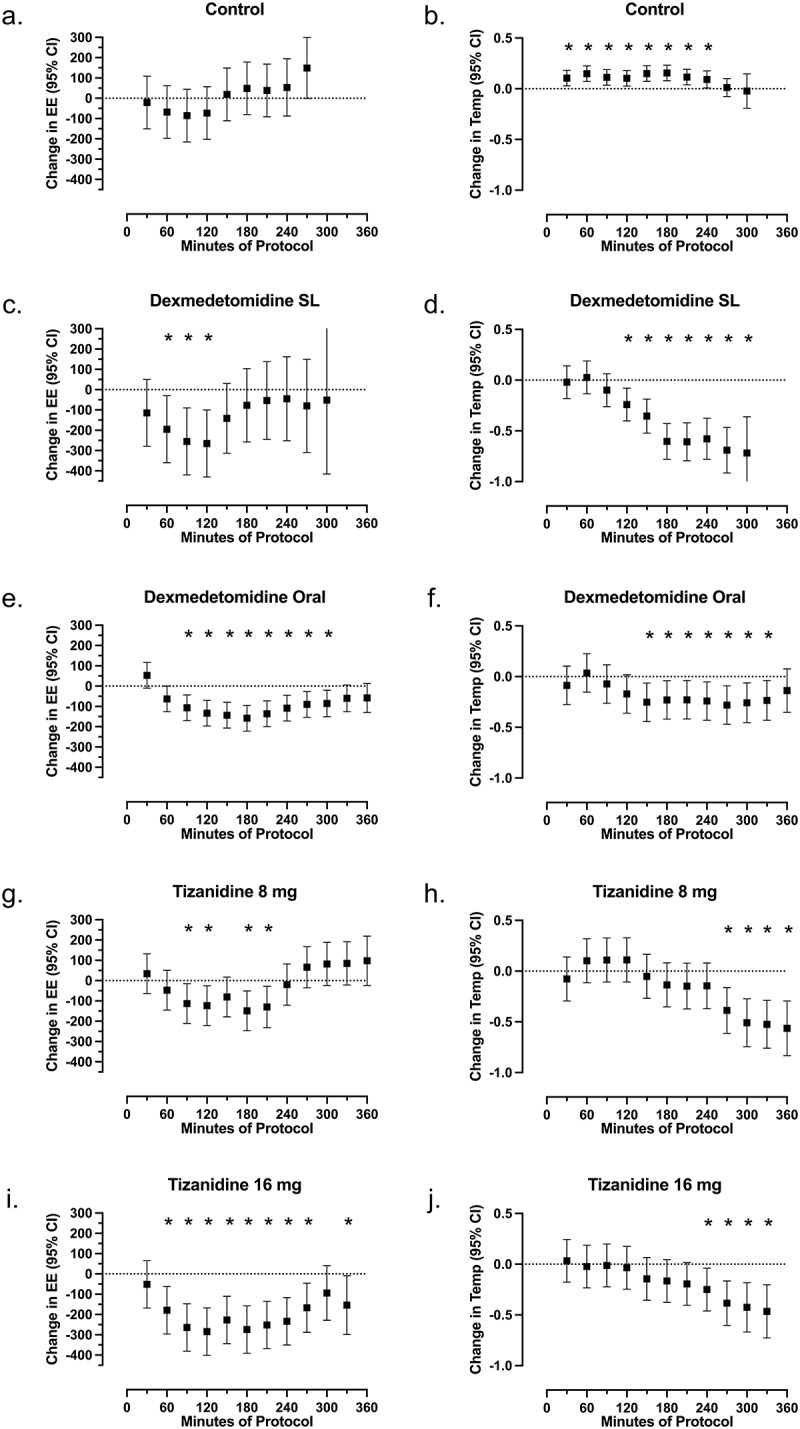
Change from baseline for energy expenditure and core temperature. Mean changes from baseline with 95% confidence intervals are plotted for energy expenditure (a, c, e and g) and core temperature (b, d, f, and h) for control (a and a), 1 µg/kg sublingual dexmedetomidine (c and d), 4 µg/kg oral dexmedetomidine (e and f), 8 mg tizanidine (g and h), and 16 mg tizanidine (i and j). Points that differ from baseline (*p* < 0.05) are indicated by a symbol (*).

After drug administration, participants reported feeling tired and sleeping for most of the monitoring period. We could easily awaken participants by voice. MANOVA detected changes over time in energy expenditure and core body temperature after every drug dose (each *p* < 0.0001). In the control group, energy expenditure was not different from baseline at any time point, and core body temperature increased relative to baseline from 30 to 240 minutes (Figure 1 (a–b)).

Changes in energy expenditure and temperature after dexmedetomidine are shown in Figure 1(c–f) and Table 2. Energy expenditure decreased after both sublingual (60–120 minutes) and oral dexmedetomidine (90–270 minutes). Core body temperature decreased after both sublingual (after 120 minutes) and oral dexmedetomidine (150–300 minutes). The maximum and mean change from baseline for other physiological variables is shown in Tables 2 and 3. Dexmedetomidine administration was associated with a decrease in VO_2_, RER, heart rate, blood pressure, cardiac stroke volume index and calculated cardiac index for both doses.

**Table 3.** Maximum and mean drug-induced change in other physiological variables.

	Baseline Value	Lowest Value	Maximum Deviation (Lowest – Baseline)	%	Mean Deviation from Baseline (0–4 hours)	%
**Heart Rate (beats/min)**						
Control	64 (59 to 72)	56 (50 to 60)	−9.3 (−14 to −5.7)	−14%	−4 (−4 to 0)	−5%
Sublingual (1 µg/kg) Dexmedetomidine	56 (52 to 62)	48 (40 to 50)	−11 (−14 to −8.5)	−20%	−5 (−8 to −4)	−9%
Oral (4 µg/kg) Dexmedetomidine	65 (63 to 69)	53 (46 to 56)	−11 (−17 to −9.6)	−17%	−5 (−8 to −4)	−10%
Tizanidine 8 mg	59 (57 to 62)	47 (44 to 52)	−11 (−15 to −7.6)	−18%	−6 (−8 to −3)	−10%
Tizanidine 16 mg	65 (61 to 70)	53 (49 to 59)	−12 (−17 to −9.3)	−20%	−6 (−8 to −5)	−11%
**Respiratory Rate (breaths/min)**						
Control	14 (14 to 16)	13 (11 to 13)	−2 (−3 to −1)	−15%	0.2 (−0.2 to 0.7)	2%
Sublingual (1 µg/kg) Dexmedetomidine	14 (13 to 15)	11 (10 to 12)	−4 (−6 to −2)	−22%	−1.2 (−3 to −0.1)	−8%
Oral (4 µg/kg) Dexmedetomidine	15 (14 to 16)	14 (13 to 15)	−1 (−3 to −0.4)	−5%	0.3 (0.0 to 0.4)	2%
Tizanidine 8 mg	15 (14 to 16)	12 (11 to 13)	−3 (−5 to −2)	−18%	−0.6 (−1.7 to −0.3)	−4%
Tizanidine 16 mg	14 (12 to 15)	12 (9 to 15)	−2 (−3 to −0.2)	−13%	−0.2 (−1.2 to 0.7)	−2%
**Systolic Blood Pressure (mmHg)**						
Control	114 (109 to 121)	109 (101 to 113)	−9 (−11 to −2)	−7%	−3 (−6 to 1)	−3%
Sublingual (1 µg/kg) Dexmedetomidine	115 (111 to 123)	102 (98 to 114)	−13 (−21 to −11)	−12%	−8 (−13 to 1)	−5%
Oral (4 µg/kg) Dexmedetomidine	131 (127 to 135)	98 (95 to 103)	−33 (−36 to −30)*	−24%	−27 (−28 to −23)*	−20%
Tizanidine 8 mg	111 (108 to 117)	98 (93 to 104)	−14 (−16 to −12)	−12%	−9 (−10 to −7)	−8%
Tizanidine 16 mg	120 (106 to 127)	96 (92 to 98)	−23 (−34 to −13)*	−20%	−9 (−29 to −7)*	−8%
**Diastolic Blood Pressure (mmHg)**						
Control	63 (60 to 77)	59 (55 to 64)	−8 (−12 to −3)	−11%	−3 (−6 to 3)	−5%
Sublingual (1 µg/kg) Dexmedetomidine	70 (69 to 78)	66 (56 to 70)	−8 (−13 to −6)	−10%	−3 (−7 to −1)	−4%
Oral (4 µg/kg) Dexmedetomidine	77 (71 to 83)	57 (56 to 58)	−21 (−24 to −15)*	−26%	−11 (−20 to −11)*	−17%
Tizanidine 8 mg	70 (65 to 73)	55 (51 to 59)	−14 (−16 to −8)	−20%	−7 (−10 to −4)	−10%
Tizanidine 16 mg	79 (65 to 87)	54 (50 to 56)	−22 (−37 to −11)*	−31%	−12 (−30 to −5)*	−17%
**SVI (ml/min/m**^**2**^)						
Control	33 (30 to 46)	31 (25 to 45)	−2 (−4 to 0)	−7%	1 (−1 to 4)	2%
Sublingual (1 µg/kg) Dexmedetomidine	56 (43 to 65)	48 (39 to 55)	−8 (−13 to −4)	−16%	2 (−2 to 5)	4%
Oral (4 µg/kg) Dexmedetomidine	56 (44 to 63)	41 (35 to 49)	−12 (−19 to −8)*	−24%	−4 (−4 to −1)	−6%
Tizanidine 8 mg	59 (54 to 70)	50 (48 to 55)	−9 (−16 to −3)	−14%	3 (1 to 9)	5%
Tizanidine 16 mg	51 (44 to 62)	40 (32 to 51)	−11 (−13 to −7)	−22%	0 (−2 to 8)	0%
**CI (l/min/m**^**2**^)						
Control	2.4 (1.8 to 3.1)	1.9 (1.5 to 3.0)	−0.2 (−0.4 to −0.1)	−12%	−0.1 (−0.2 to 0.2)	−5%
Sublingual (1 µg/kg) Dexmedetomidine	3.5 (3.0 to 3.8)	2.4 (2.2 to 3.1)	−0.9 (−1.0 to −0.5)	−22%	−0.4 (−0.6 to −0.1)	−8%
Oral (4 µg/kg) Dexmedetomidine	3.7 (2.9 to 3.9)	2.4 (2.2 to 2.7)	−1.0 (−1.2 to −0.6)*	−28%	−0.5 (−0.7 to −0.3)	−14%
Tizanidine 8 mg	3.5 (3.1 to 4.1)	2.8 (2.7 to 2.9)	−0.6 (−1.3 to −0.3)	−18%	−0.2 (−0.4 to 0.1)	−5%
Tizanidine 16 mg	3.4 (2.9 to 3.9)	2.4 (2.2 to 2.8)	−1.1 (−1.2 to −0.9)*	−29%	−0.5 (−0.6 to 0.0)	−13%

Per-subject median (IQR) values for baseline, minima and change in the 30-minute averages for each physiological variable. Maximum deviation is zero when the baseline value was the lowest recorded value. % Change is the median maximum absolute decrease in real variables. A symbol (*) indicates *p* < 0.05 for comparison of drug group relative to control.

Changes in energy expenditure and temperature after tizanidine are shown in Figure 1(g–j) and Table 2. Energy expenditure decreased after both 8 mg tizanidine (90–120 and 180–210 minutes) and 16 mg tizanidine (60–270 minutes). Core body temperature decreased after both 8 mg tizanidine (after 270 minutes) and 16 mg tizanidine (after 240 minutes). The maximum and mean change from baseline for other physiological variables is shown in Tables 2 and 3. Tizanidine administration was associated with a decrease in VO_2_, RER, heart rate, blood pressure, cardiac stroke volume index and calculated cardiac index for both doses.

### Comparison between drug groups

We detected drug group effects on maximum decrease in systolic blood pressure (F_4,39_ = 9.97; *p* < 0.0001), diastolic blood pressure (F_4,39_ = 5.22; *p* = 0.0021), stroke volume index (F_4,39_ = 3.22; *p* = 0.0236) and calculated cardiac index (F_4,39_ = 4.51; *p* = 0.0048). We detected drug group effects on mean deviation in core body temperature (F_4,39_ = 5.27; *p* = 0.0019), systolic blood pressure (F_4,39_ = 11.06; *p* < 0.0001) and diastolic blood pressure (F_4,39_ = 5.95; *p* = 0.0009).

The maximum decrease in systolic blood pressure (*p* < 0.001), diastolic blood pressure (*p* = 0.019), cardiac stroke volume index (*p* = 0.019), and calculated cardiac index (*p* = 0.009) differed from control after oral dexmedetomidine. The mean change from baseline for core body temperature differed from control after sublingual (*p* = 0.001) and oral dexmedetomidine (*p* = 0.026). Mean change from baseline in systolic (*p* < 0.001) and diastolic blood pressure (*p* = 0.01) differed from control after oral dexmedetomidine.

The maximum decrease in systolic blood pressure (*p* = 0.013), diastolic blood pressure (*p* = 0.017), and calculated cardiac index (*p* = 0.007) differed from control after 16 mg tizanidine. Mean change from baseline in systolic (*p* = 0.005) and diastolic blood pressure (*p* = 0.008) differed from control after 16 mg tizanidine.

### Associations between physiological changes and core temperature

**Figure 2.**
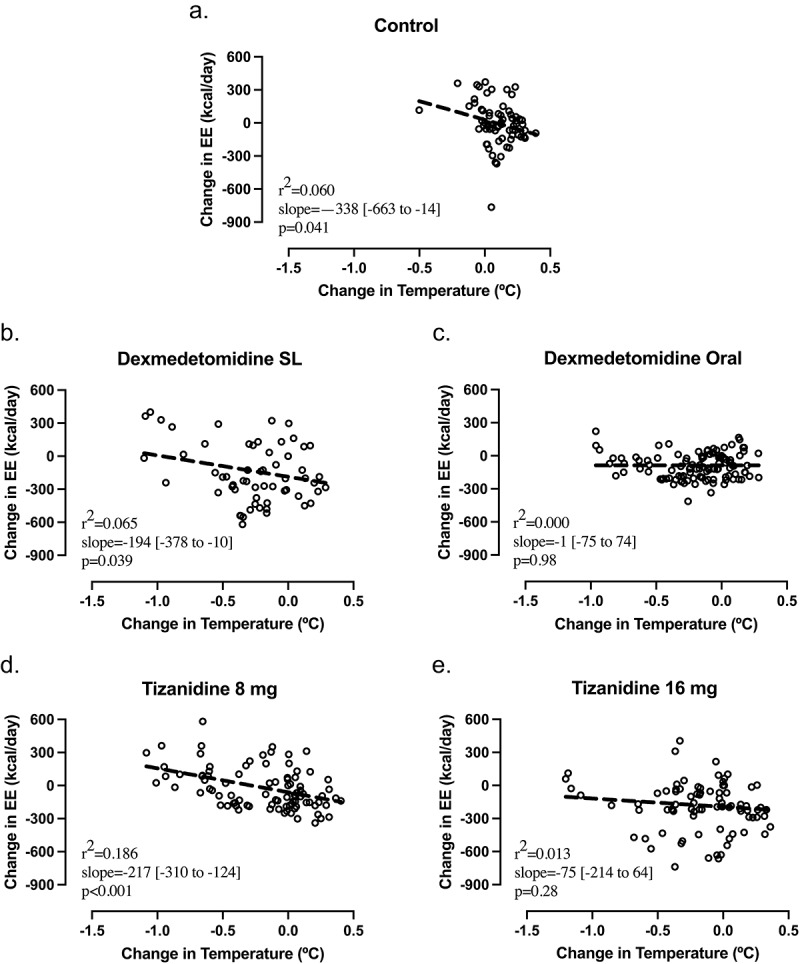
Associations between change in energy expenditure (EE) and change in core temperature. Values for each 30-minute epoch are plotted with ordinary linear regression lines. Reduction in core temperature were associated with smaller decreases in EE in (a) control conditions (*n* = 8, 5 male: 3 female) (a) and after (b) sublingual (SL) dexmedetomidine (*n* = 8, 6 male: 2 female) and (d) 8 mg tizanidine (*n* = 8, 7 male: 1 female), but not after (c) oral dexmedetomidine (*n* = 9, 4 male: 5 female) or (e) 16 mg tizanidine (*n* = 8, 6 male: 2 female).

Associations between change in energy expenditure and change in core temperature are shown for control and for each drug in Figure 2. The negative slope of regression lines in Figure 2(a,b) and (d) indicates that change in energy expenditure decreased as change in temperature increased under control conditions and after sublingual dexmedetomidine or 8 mg oral tizanidine. The flat slope of regression lines in Figure 2(c) and (e) indicates that there was no detectable increase in energy expenditure with decrease in temperature after oral dexmedetomidine or 16 mg tizanidine.

The association of changes in other physiological variables with changes in energy expenditure or core temperature are shown in Table 4. Changes in all variables except respiratory rate were proportional to changes in energy expenditure after 16 mg tizanidine. Change in respiratory rate, blood pressure, stroke volume index, and cardiac index were related to energy expenditure after oral dexmedetomidine. Changes in all variables except stroke volume index were related to changes in temperature after oral dexmedetomidine. Blood pressure was negatively correlated with temperature after tizanidine.

**Table 4.** Association between changes in physiological variables.

	Control		Sublingual (1 µg/kg) Dexmedetomidine		Oral (4 µg/kg) Dexmedetomidine		Tizanidine 8 mg		Tizanidine 16 mg	
	R	p-value	R	p-value	R	p-value	R	p-value	R	p-value
**Change in Energy Expenditure**										
∆HR	0.551	<0.0001	−0.311	0.007	0.0143	0.88	0.419	<0.0001	0.502	<0.0001
∆RR	−0.102	0.38	0.142	0.23	0.265	0.004	0.102	0.31	−0.443	0.0002
∆SBP	−0.022	0.85	0.522	<0.0001	0.138	0.14	0.564	<0.0001	0.503	<0.0001
∆DBP	0.144	0.21	0.277	0.026	0.345	0.0001	0.650	<0.0001	0.413	0.0001
∆SVI	−0.096	0.43	0.067	0.57	0.483	<0.0001	−0.075	0.46	0.495	<0.0001
∆CI	0.093	0.44	0.155	0.19	0.418	<0.0001	0.196	0.052	0.579	<0.0001
**Change in Temperature**										
∆HR	0.088	0.44	−0.150	0.21	0.234	0.01	0.382	0.0001	0.089	0.41
∆RR	0.130	0.25	0.359	0.002	−0.208	0.023	0.046	0.65	0.277	0.022
∆SBP	−0.037	0.75	0.202	0.13	0.237	0.0096	−0.482	<0.0001	−0.427	<0.0001
∆DBP	−0.110	0.34	0.290	0.03	0.366	<0.0001	−0.475	<0.0001	−0.482	<0.0001
∆SVI	−0.098	0.41	0.156	0.19	−0.002	0.98	0.123	0.23	−0.170	0.11
∆CI	−0.221	0.06	0.433	0.002	0.199	0.044	0.399	<0.0001	0.025	0.82

Correlation coefficients (R) from simple linear regression of change in energy expenditure or temperature versus change in heart rate (∆HR), respiratory rate (∆RR), systolic blood pressure (∆SBP), diastolic blood pressure (∆DBP), stroke volume index (∆SVI), and cardiac index (∆CI). P-values are uncorrected.

### Comparison of physiological changes with drug concentrations

**Figure 3.**
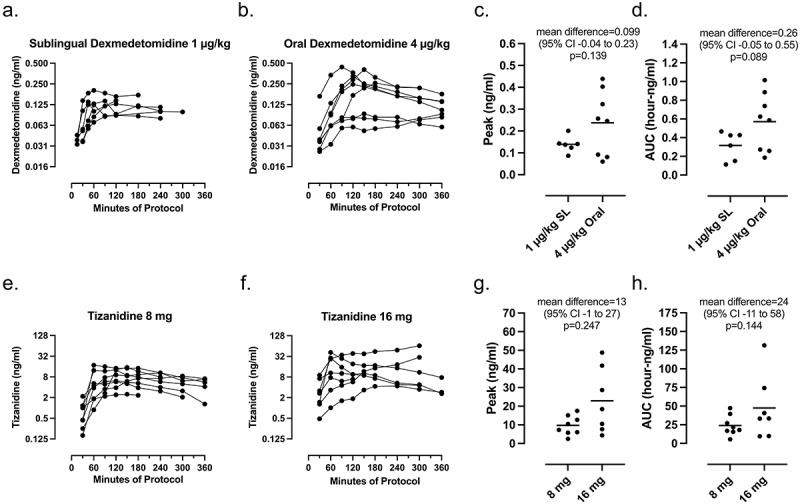
Time course of plasma concentrations for each drug in individual participants. Samples could not be obtained due to difficulties drawing from the intravenous catheter for 2 participants after sublingual dexmedetomidine, for 1 participant after oral dexmedetomidine, and for 1 participant after 16 mg tizanidine.

Plasma drug AUC within experiment was not associated with mean change in energy expenditure for sublingual dexmedetomidine, oral dexmedetomidine, 8 mg tizanidine or 16 mg tizanidine (r^2^ = 0.26, 0.01, 0.03, 0.001 respectively) (Figure 3). Plasma drug level was not associated with mean change in temperature for sublingual dexmedetomidine, 8 mg tizanidine or 16 mg tizanidine, (r^2^ = 0.00, 0.12, 0.38 respectively), but was associated with change in temperature for oral dexmedetomidine (r^2^ = 0.61, *p* = 0.03). Three participants had particularly low peak and AUC for plasma drug levels after oral dexmedetomidine. These participants were all female, but this may be a chance association with such small numbers.

## Discussion

This study found that single oral doses of both dexmedetomidine and tizanidine reduced energy expenditure relative to the baseline resting energy expenditure even in the setting of external cooling. Control group energy expenditure did not decline during sessions without drug administration (Figure 1), perhaps because experiments began in the late morning when human energy expenditure rates are increasing [28,29]. This drug-induced reduction in energy expenditure is in excess of the decrease attributable to resting quietly, because all participants had similar quiet rest and reported light sleep during baseline. The change in energy expenditure over time shows an onset and offset consistent with drug action (Figures 2(b,f)) and 3(b,f), even though participants were lying quietly during the entire session. Furthermore, the minimum energy expenditure after dexmedetomidine or tizanidine (Table 2) was below the pREE predicted by age, sex, height, and mass (Table 1), which is also consistent with a drug effect. These data confirm previous studies that used intravenous dexmedetomidine to facilitate induction of hypothermia by inhibiting the shivering response [14–17,30,31]. These changes are similar to the effects of oral clonidine, another A2AR agonist that produces peak sedation about 2 hours after ingestion [32] and that reduces energy expenditure for at least 180 minutes [33].

The oral drugs at the doses used in this study did not completely eliminate shivering in response to the strong stimulus from the external cooling device, although only a few participants in each group exhibited overt shivering on the clinical scale. Because we immediately adjusted the cooling device when shivering appeared, we cannot make comparisons of shivering intensity or frequency with a constant stimulus. Prior experiments have demonstrated that shivering during forced cooling can be suppressed with intravenous drugs [14,15,34] and that shivering increases metabolic rate [34–36]. In fact, increasing energy expenditure is probably a more sensitive and direct measure than the clinical scale for detecting the thermoregulatory response to cooling [6,25].

In this study, the negative slope of the regression line in (Figure 2(b,d)) shows that the increase in energy expenditure with declining temperature was present after 1 µg/kg sublingual dexmedetomidine and 8 mg of oral tizanidine. However, the flat regression line in (Figure 2(c,e)) suggests that this cooling-induced increase was inhibited after 4 µg/kg oral dexmedetomidine and 16 mg of oral tizanidine. Thus, the higher doses of these drugs may be more useful to control shivering and cold-induced thermogenesis.

Both dexmedetomidine and tizanidine affected other physiological variables. Heart rate, blood pressure, respiratory rate, cardiac stroke volume index, and cardiac index declined. These decreases were associated with the change in metabolic rate after higher drug doses (Table 4). Blood pressure declined with declining temperature after dexmedetomidine, but this relationship was reversed for tizanidine. This is consistent with reports that that dexmedetomidine suppresses a cold-pressor reflex [37] and suggests that tizanidine does not share this effect. Respiratory rate changes were not clinically significant, and participants had no hypoxemia. While the monitoring system did not permit end-tidal CO_2_ measurement in these experiments, previous studies found no clinically significant impairment of resting ventilation after intravenous dexmedetomidine [37–41]. The reduction of heart rate and cardiac index are consistent with drug-induced sympatholysis, which also reduces systemic resistance and myocardial demand [42].

The plasma drug levels provide information about using these novel routes for dexmedetomidine and these higher doses of tizanidine. Sublingual dexmedetomidine resulted in much more consistent plasma concentrations than oral dexmedetomidine (Figure 3). In fact, peak and AUC drug levels for three participants after oral dexmedetomidine were less than peak and AUC levels for any participant receiving sublingual drug, perhaps indicating enteral breakdown of drug or prolonged enteral absorption for some individuals. Our participants achieved peak plasma levels that were just at the minimum levels associated with sedation in prior studies (0.2 to 0.3 ng/ml) and well below levels associated with deep sedation (>1.9 ng/ml) [37]. Consistent with more protracted absorption, peak levels were much lower than reported or expected with intravenous bolus of similar doses [14,43,44]. These data suggest that a sublingual or mucosal route for dexmedetomidine might provide reliable bolus delivery with less risk for high-dose toxicity than intravenous bolus. These pharmacokinetic data about dexemedetomidine may inform other uses for this drug, such as anesthetic premedication or treatment of agitation [45,46].

Plasma tizanidine levels were similar to those after 16 mg oral doses in other studies that were reported to reliably produce a side-effect of drowsiness (10–20 ng/ml) [47]. The peak and AUC tizanidine plasma levels varied between individuals, particularly after the higher dose. The longer time to peak levels and the more sustained levels of tizanidine relative to dexmedetomidine (Figure 3) may make this drug useful for long-term treatment but more difficult to titrate in short-term experiments. The drug levels measured in these experiments only examined the absorption and peak levels for each drug. We did not attempt to collect delayed blood samples as would be required to estimate clearance, which has been studied extensively in prior studies [37,47].

Most physiological effects of the drugs were short-lived relative to plasma drug levels, and plasma drug levels were weakly correlated with physiological effects within experiments. Participants also reported recovery from the subjective sedative effects of the drug by the end of monitoring, although plasma levels were still detectable. This may reflect that drug effects are more related to levels in the central nervous system, which we did not measure, rather than levels in plasma. Alternatively, participants may develop some tachyphylaxis to drug effects. Selection of drug doses and design of dosing regimens for longer duration manipulation of metabolic rate and temperature will need to empirically test drug doses or titrate dosing to observed effect.

The reduction of temperature after drug administration in this study appears to be a consequence of reduced energy expenditure rather than a cause of reduced energy expenditure. Core temperature declined in participants during the study period beginning 60–120 minutes after peak reduction of energy expenditure (Figure 1). This time lag suggests that reduced metabolic rate facilitated cooling. In many participants, the largest temperature reductions occurred near the end of observation when energy expenditure was returning to baseline. The negative slopes of regression lines for energy expenditure versus temperature in Figure 2(a,b) and (d) are also consistent with participants having cold-induced thermogenesis in control conditions and after the lower doses of dexmedetomidine and tizanidine. Cold-induced increase in energy expenditure may offset any drug-induced reduction in energy expenditure.

These results inform strategies for developing a protocol for long-duration metabolic reduction. Our results indicate that the lower doses of dexmedetomidine and tizanidine did not reduce the critical temperature for triggering cold-induced thermogenesis as much as intravenous dexmedetomidine in prior studies [14,17,30,31]. However, the consistent reduction of energy expenditure even when temperature decreased after higher dose of dexmedetomidine and tizanidine (Figure 2) suggests that these drug doses did blunt cold-induced thermogenesis. Sustained plasma drug levels similar to or higher than those achieved here may be useful for prolonged reduction of energy expenditure in humans. If drug-induced and hypothermia-induced reduction in energy expenditure [17] are additive, larger reductions may be possible if drug levels are prolonged long enough to achieve significant reduction of temperature without cold-induced thermogenesis.

Reducing energy expenditure in humans has several potential applications. For example, these drugs might reduce the total food consumption, oxygen consumption and carbon dioxide removal required during long-duration spaceflight [10]. Such a reduction would reduce payload and prolong possible mission duration. Concomitant sedation would also reduce psychological distress from prolonged confinement. Energy expenditure reduction and resource conservation also could serve as a strategy to prolong survival time during rescues from confined spaces such as a submarine, cave or even a disabled spacecraft. Other applications of metabolic rate reduction include protection of organs from ischemia, hypoperfusion, hypoxemia [11–13] or radiation [48]. Temperature control or even reduced temperature is potentially beneficial for acquired brain injury, sepsis or other critical illnesses [13,49]. These data and the prior literature suggest that A2AR agonist drugs can be useful to inhibit shivering or lower energy expenditure in these critical care settings. However, the effects of these drugs on multiple physiological parameters will require that each be carefully tested in each clinical setting. For example, in head injured patients, intravenous clonidine reduced perfusion pressure and had variable effects on cerebral oxygen extraction [50]. Developing methods to manipulate metabolic rate in spontaneously breathing people will allow study of more applications.

Preclinical studies have explored the mechanism of action for dexmedetomidine and tizanidine. Both dexmedetomidine and tizanidine bind to and activate A2ARs but have negligible affinity or activity at beta-adrenergic receptors [51] and orders of magnitude lower affinity and activity at alpha-1 receptors [52]. A2ARs in the central nervous system include both autoreceptors that reduce norepinephrine release from noradrenergic neurons and heteroreceptors that reduce other neurotransmitter release from non-adrenergic neurons [53]. Studies with transgenic mice suggest that both alpha-2 autoreceptors and heteroreceptors are involved with sedation, but that alpha-2 heteroreceptors are most important for effects on temperature regulation [54]. Sedative effects of dexmedetomidine in animals are related to activity in brain regions including the locus coeruleus, tuberomammillary nucleus and ventrolateral preoptic nucleus of the hypothalamus [55]. Lesion studies and direct drug injection studies implicate noradrenergic innervation of the medial preoptic area of brain with temperature regulation [56]. A2AR agonists reduced metabolic rate, and the A2AR antagonist idazoxan increased metabolic rate in cattle [57]. Both tizanidine and dexmedetomidine can reduce plasma catecholamine levels [37,58]. This effect could reduce thermogenesis and energy expenditure mediated by circulating catecholamines [59].

These drugs also may influence energy consumption and shivering by reducing skeletal muscle activity. Tizanidine affects the activity of spinal motoneurons and muscle activity both through A2AR effects and also through actions on imidazoline receptors [60]. For example, tizanidine inhibition of spinal reflexes in rats is blocked more by the combined A2AR and imidazole receptor-binding drug idazoxan than by the A2AR antagonist yohimbine [61]. Tizanidine also inhibits spinal reflexes when injected directly into the brain and is dependent on both brain catecholamines and imidazole receptors [62]. This suggests that tizanidine inhibition of reflexes may be mediated by reduction in noradrenergic facilitation of spinal motoneuron activity.

Limitations of this study included the small proportion of female participants, lack of blinding and absence of a placebo in the control group. Given the small numbers of women, this study lacks statistical power to compare variables between sexes. However, the inclusion of both sexes does increase the generalizability of results. We did not blind participants or investigators as a safety measure in this study of off-label drug use. This decision was also based on the fact that our outcome measures were all physiological variables collected by automated monitors. Subjective bias is less likely to influence these observed outcomes compared to participant-reported measures. Participants took dexmedetomidine by mouth in less than 4 ml of saline vehicle and took tizanidine by tablets which contained only inert solids in addition to drug. Therefore, it is implausible that addition of a placebo to the control group would have added any nonspecific physiological effects.

Another limitation of this study is that the magnitude of temperature change achieved in this study may have been too small to detect temperature-related reductions in energy expenditure. An 8–10% reduction in VO_2_ per 1°C reduction in temperature is often cited and may occur during reduction of fever with shivering [13,63]. About 6% per 1°C reduction in cerebral oxygen utilization in anesthetized, non-shivering adults occurs with cooling from 37°C to 30°C [8]. In a previous study using continuous intravenous infusions of dexmedetomidine during induction of hypothermia to prevent shivering, we found a temperature-dependent reduction of VO_2_ of 5.6%/ºC in the range from 37°C to 33°C [17]. Because most participants in this study had <1°C temperature change, the observed 13% to 22% peak decrease in energy expenditure is too large to attribute to temperature-dependent changes (Table 2). The changes in temperature in the present study are also too small to determine if drug-induced reduction in energy expenditure and temperature-dependent reduction in energy expenditure are additive.

The magnitude and rate of temperature reduction after drug ingestion may be specific to the laboratory conditions. We placed a surface cooling device that covered 8–15% of total body surface area on the back of each participant to ensure there was opportunity for net heat loss during the experiment. We think this device was necessary to observe decreases in core temperature because the canopy and coverings for metabolic measurements create a blanket insulating the anterior part of the body. Core temperature may not change as much if participants had less active heat removal or were in warmer environments that maintained neutral heat balance [6]. Likewise, temperature changes might be greater if cooling pads were covering a larger proportion of body surface area or if ambient temperature was lower. Future studies should monitor heat flux in various body regions to provide better understanding of the balance between heat production and loss.

Finally, our primary analysis is a within-subject statistical comparison of baseline versus post-drug epochs rather than contrast with control. This approach maximized statistical power while reducing participant burden. Although this study lacked statistical power to compare marginal effects across drugs and doses, we were able to detect group differences in the maximal change in blood pressure and cardiac indices, as well as in the mean change in temperature and blood pressure. Though statistically weak, the absolute values of physiological effects also increased with drug dose (Tables 2 and 3, Figure 3), further supporting that these are real drug effects.

In summary, we confirmed that oral dexmedetomidine and tizanidine reduce energy expenditure during external cooling for several hours in healthy humans. This reduction is associated with mild sedation from which it is easy to arouse participants and is independent of reductions in core body temperature that begin about 60–120 minutes after peak reduction of energy expenditure. Higher doses of oral dexmedetomidine and oral tizanidine suppress cold-induced thermogenesis, which can facilitate induced cooling. Dosing regimens that provide sustained levels of these drugs in the range observed after the higher doses in this study, may allow induction of metabolic suppression and hypothermia in humans.

## Abbreviations

A2AR: alpha-2-adrenergic receptor
ANOVA: analysis of variance
AUC: area under the curve
AV: atrioventricular
BSAS: bedside shivering assessment scale
CI: cardiac index
EDTA: ethylenediaminetetraacetic acid
E: energy expenditure
ESI: electrospray ionization
ESS: Epworth Sleepiness Scale
FDA: Food and Drug Administration
FECO_2_: fraction expired carbon dioxide
HCG: human chorionic gonadotropin
HPLC: high-performance liquid chromatography
LC-MS/MS: liquid chromatography-mass spectrometry/mass spectrometry
m/z: mass to charge ratio
MANOVA: multiple variable analysis of variance
pREE: predicted resting energy expenditure
RER: respiratory exchange ratio
SD: standard deviation
SpO_2_: Saturation of oxygen by pulse oximetry
SRM: selected reaction monitoring
SVI: stroke volume index
UPLC: ultra-performance liquid chromatography
VO_2_: Rate of oxygen consumption
VCO_2_: Rate of carbon dioxide production

## References

[1] Ruf T, Geiser F. Daily torpor and hibernation in birds and mammals. Biol Rev Camb Philos Soc. 2015;90(3):891–926. doi: 10.1111/brv.12137 25123049 PMC4351926

[2] Tøien Ø, Blake J, Edgar DM, et al. Hibernation in black bears: independence of metabolic suppression from body temperature. Science. 2011;331(6019):906–909. doi: 10.1126/science.1199435 21330544

[3] Geiser F, Kenagy GJ. Torpor duration in relation to temperature and metabolism in hibernating ground squirrels. Physiol Zool. 1988;61(5):442–449. doi: 10.1086/physzool.61.5.30161266.

[4] Davis DE. Hibernation and circannual rhythms of food consumption in marmots and ground squirrels. Q Rev Biol. 1976;51(4):477–514. doi: 10.1086/409594 799318

[5] Scholander PF, Hock R, Walters V, et al. Heat regulation in some arctic and tropical mammals and birds. Biol Bull. 1950;99(2):237–258. doi: 10.2307/1538741 14791422

[6] Romanovsky AA. The thermoregulation system and how it works. Handb Clin Neurol. 2018;156:3–43. doi: 10.1016/B978-0-444-63912-7.00001-1 30454596

[7] Geiser F. Metabolic rate and body temperature reduction during hibernation and daily torpor. Annu Rev Physiol. 2004;66(1):239–274. doi: 10.1146/annurev.physiol.66.032102.115105 14977403

[8] McCullough JN, Zhang N, Reich DL, et al. Cerebral metabolic suppression during hypothermic circulatory arrest in humans. Ann Thorac Surg. 1999;67(6):1895–1899. doi: 10.1016/s0003-4975(99)00441-5 10391334

[9] Abjigitova D, Notenboom ML, Veen KM, et al. Optimal temperature management in aortic arch surgery: a systematic review and network meta-analysis. J Card Surg. 2022;37:5379–5387. doi: 10.1111/jocs.17206 36378895 PMC10098497

[10] Regan MD, Flynn-Evans EE, Griko YV, et al. Shallow metabolic depression and human spaceflight: a feasible first step. J Appl Physiol (1985). 2020;128(3):637–647. doi: 10.1152/japplphysiol.00725.2019 31999524 PMC7099441

[11] Villablanca PA, Rao G, Briceno DF, et al. Therapeutic hypothermia in ST elevation myocardial infarction: a systematic review and meta-analysis of randomised control trials. Heart. 2016;102(9):712–719. doi: 10.1136/heartjnl-2015-308559 26864673

[12] Kuczynski AM, Marzoughi S, Al Sultan AS, et al. Therapeutic hypothermia in acute ischemic stroke—a systematic review and meta-analysis. Curr Neurol Neurosci Rep. 2020;20(5):13. doi: 10.1007/s11910-020-01029-3 32372297

[13] Petitjeans F, Leroy S, Pichot C, et al. Hypothesis: fever control, a niche for alpha-2 agonists in the setting of septic shock and severe acute respiratory distress syndrome? Temperature. 2018;5(3):3, 224–256. doi: 10.1080/23328940.2018.1453771 PMC6209424 30393754

[14] Callaway CW, Elmer J, Guyette FX, et al. Dexmedetomidine reduces shivering during mild hypothermia in waking subjects. PLOS ONE. 2015;10(8):e0129709. doi: 10.1371/journal.pone.0129709 26237219 PMC4523180

[15] Rittenberger JC, Flickinger KL, Weissman A, et al. Cooling to facilitate metabolic suppression in healthy individuals. Aerosp Med Hum Perform. 2019;90(5):475–479. doi: 10.3357/AMHP.5284.2019 31023408 PMC7077737

[16] Rittenberger JC, Weissman A, Flickinger KL, et al. Glycopyrrolate does not ameliorate hypothermia associated bradycardia in healthy individuals: a randomized crossover trial. Resuscitation. 2021;164:79–83. doi: 10.1016/j.resuscitation.2021.05.020 34087418 PMC8259805

[17] Flickinger KL, Weissman A, Jonathan Elmer J, et al. Metabolic manipulation and therapeutic hypothermia. Ther Hypothermia Temp Manag. 2023;14(1):46–51. In press. doi: 10.1089/ther.2023.0010. 37405749

[18] Scharf MT. Reliability and efficacy of the epworth sleepiness scale: is there still a place for it? Nat Sci Sleep. 2022;14:2151–2156. doi: 10.2147/NSS.S340950 36536636 PMC9759004

[19] American College of Sports Medicine. ACSM’s guidelines for exercise testing and prescription. 8th ed. Baltimore, MD: Walters-Kluwer, Lippincott Williams and Wilkins; 2010.

[20] Massy-Westropp NM, Gill TK, Taylor AW, et al. Hand grip strength: age and gender stratified normative data in a population-based study. BMC Res Notes. 2011 Apr 14;4(1):127. PMID: 21492469; PMCID: PMC3101655. 21492469 10.1186/1756-0500-4-127 PMC3101655

[21] Mifflin MD, St Jeor ST, Hill LA, et al. A new predictive equation for resting energy expenditure in healthy individuals. Am J Clin Nutr. 1990;51(2):241–247. doi: 10.1093/ajcn/51.2.241 2305711

[22] Weir JB. New methods for calculating metabolic rate with special reference to protein metabolism. J Physiol. 1949;109(1–2):1–9. doi: 10.1113/jphysiol.1949.sp004363 15394301 PMC1392602

[23] Raval NY, Squara P, Cleman M, et al. Multicenter evaluation of noninvasive cardiac output measurement by bioreactance technique. J Clin Monit Comput. 2008;22(2):113–119. doi: 10.1007/s10877-008-9112-5 18340540

[24] Rich JD, Archer SL, Rich S. Noninvasive cardiac output measurements in patients with pulmonary hypertension. Eur Respir J. 2013;42(1):125–133. doi: 10.1183/09031936.00102212 23100501

[25] Badjatia N, Strongilis E, Gordon E, et al. Metabolic impact of shivering during therapeutic temperature modulation: the bedside shivering assessment scale. Stroke. 2008;39(12):3242–3247. doi: 10.1161/STROKEAHA.108.523654 18927450

[26] Birabaharan J, RE W 3rd, Nolin TD, et al. Simultaneous detection of a panel of nine sedatives and metabolites in plasma from critically ill pediatric patients via UPLC-MS/MS. J Pharm Biomed Anal. 2022;218:114853. doi: 10.1016/j.jpba.2022.114853 35659658 PMC9302904

[27] Guidance for Industry – Bioanalytical Method Validation, U.S. Food and Drug Administration. 2018 May [cited February 22, 2023]. Available from: https://www.fda.gov/downloads/drugs/guidances/ucm070107.pdf

[28] Ruddick-Collins LC, Flanagan A, Johnston JD, et al. Circadian rhythms in resting metabolic rate account for apparent daily rhythms in the thermic effect of food. J Clin Endocrinol Metab. 2022;107(2):e708–e715. doi: 10.1210/clinem/dgab654 34473293 PMC8764350

[29] Zitting KM, Vujovic N, Yuan RK, et al. Human resting energy expenditure varies with circadian phase. Curr Biol. 2018;28(22):3685–3690.e3.e3. doi: 10.1016/j.cub.2018.10.005 30416064 PMC6300153

[30] Lenhardt R, Orhan-Sungur M, Komatsu R, et al. Suppression of shivering during hypothermia using a novel drug combination in healthy volunteers. Anesthesiology. 2009;111(1):110–115. doi: 10.1097/ALN.0b013e3181a979a3 19512867

[31] Talke P, Tayefeh F, Sessler DI, et al. Dexmedetomidine does not alter the sweating threshold, but comparably and linearly decreases the vasoconstriction and shivering thresholds. Anesthesiology. 1997;87(4):835–841. doi: 10.1097/00000542-199710000-00017 9357885

[32] Dollery CT, Davies DS, Draffan GH, et al. Clinical pharmacology and pharmacokinetics of clonidine. Clin Pharmacol Ther. 1976;19(1):11–17. doi: 10.1002/cpt197619111 1245090

[33] Takahashi H, Nishikawa T, Mizutani T, et al. Oral clonidine premedication decreases energy expenditure in human volunteers. Can J Anaesth. 1997;44(3):268–272. doi: 10.1007/BF03015364 9067045

[34] Hostler D, Northington WE, Callaway CW. High-dose diazepam facilitates core cooling during cold saline infusion in healthy volunteers. Appl Physiol Nutr Metab. 2009;34(4):582–586. doi: 10.1139/H09-011 19767791

[35] Acosta FM, Martinez-Tellez B, Sanchez-Delgado G, et al. Physiological responses to acute cold exposure in young lean men. PLOS ONE. 2018;13(5):e0196543. doi: 10.1371/journal.pone.0196543 29734360 PMC5937792

[36] Taniguchi Y, Lenhardt R, Sessler DI, et al. The effect of altering skin-surface cooling speeds on vasoconstriction and shivering thresholds. Anesth Analg. 2011;113(3):540–544. doi: 10.1213/ANE.0b013e3182273b19 21778332

[37] Ebert TJ, Hall JE, Barney JA, et al. The effects of increasing plasma concentrations of dexmedetomidine in humans. Anesthesiology. 2000;93(2):382–394. doi: 10.1097/00000542-200008000-00016 10910487

[38] Belleville JP, Ward DS, Bloor BC, et al. Effects of intravenous dexmedetomidine in humans. I. Sedation, ventilation, and metabolic rate. Anesthesiology. 1992 Dec;77(6):1125–1133. 10.1097/00000542-199212000-00013 PMID: 1361310 1361310

[39] Hsu YW, Cortinez LI, Robertson KM, et al. Dexmedetomidine pharmacodynamics: part I: crossover comparison of the respiratory effects of dexmedetomidine and remifentanil in healthy volunteers. Anesthesiology. 2004;101(5):1066–1076. doi: 10.1097/00000542-200411000-00005 15505441

[40] Venn RM, Hell J, Grounds RM. Respiratory effects of dexmedetomidine in the surgical patient requiring intensive care. Crit Care. 2000;4(5):302–308. doi: 10.1186/cc712 11056756 PMC29047

[41] Lodenius Å, Ebberyd A, Hårdemark Cedborg A, et al. Sedation with Dexmedetomidine or Propofol impairs hypoxic control of breathing in healthy male volunteers: a nonblinded, randomized crossover study. Anesthesiology. 2016;125(4):700–715. doi: 10.1097/ALN.0000000000001236 27483127

[42] Snapir A, Posti J, Kentala E, et al. Effects of low and high plasma concentrations of dexmedetomidine on myocardial perfusion and cardiac function in healthy male subjects. Anesthesiology. 2006;105(5):902–910. doi: 10.1097/00000542-200611000-00010 17065883

[43] Iirola T, Vilo S, Manner T, et al. Bioavailability of dexmedetomidine after intranasal administration. Eur J Clin Pharmacol. 2011;67(8):825–831. doi: 10.1007/s00228-011-1002-y 21318594

[44] Weerink MAS, Struys MMRF, Hannivoort LN, et al. Clinical pharmacokinetics and pharmacodynamics of dexmedetomidine. Clin Pharmacokinet. 2017;56(8):893–913. doi: 10.1007/s40262-017-0507-7 28105598 PMC5511603

[45] Xiong J, Gao J, Pang Y, et al. Dexmedetomidine premedication increases preoperative sedation and inhibits stress induced by tracheal intubation in adult: a prospective randomized double-blind clinical study. BMC Anesthesiol. 2022;22(1):398. doi: 10.1186/s12871-022-01930-z 36544098 PMC9768986

[46] Preskorn SH, Zeller S, Citrome L, et al. Effect of sublingual dexmedetomidine vs Placebo on acute agitation associated with bipolar disorder: a randomized clinical trial. JAMA. 2022;327(8):727–736. doi: 10.1001/jama.2022.0799 35191924 PMC8864508

[47] Henney, Runyan JD, Henney HR, et al. A clinically relevant review of tizanidine hydrochloride dose relationships to pharmacokinetics, drug safety and effectiveness in healthy subjects and patients. Int J Clin Pract. 2008;62(2):314–324. doi: 10.1111/j.1742-1241.2007.01660.x 18199279

[48] Puspitasari A, Squarcio F, Quartieri M, et al. Synthetic torpor protects rats from exposure to accelerated heavy ions. Sci Rep. 2022;12(1):16405. doi: 10.1038/s41598-022-20382-6 36180516 PMC9525701

[49] Elmer J, Callaway CW. Temperature control after cardiac arrest. Resuscitation. 2023;189:109882. doi: 10.1016/j.resuscitation.2023.109882 37355091 PMC10530429

[50] Ter Minassian A, Beydon L, Decq P, et al. Changes in cerebral hemodynamics after a single dose of clonidine in severely head-injured patients. Anesth Analg. 1997;84(1):127–132. doi: 10.1213/00000539-199701000-00024 8989013

[51] Proudman RGW, Akinaga J, Baker JG. The signaling and selectivity of α-adrenoceptor agonists for the human α2A, α2B and α2C-adrenoceptors and comparison with human α1 and β-adrenoceptors. Pharmacol Res Perspect. 2022;10(5):e01003. doi: 10.1002/prp2.1003 36101495 PMC9471048

[52] Proudman RGW, Baker JG. The selectivity of α-adrenoceptor agonists for the human α1A, α1B, and α1D-adrenoceptors. Pharmacol Res Perspect. 2021;9(4):e00799. PMID: 34355529. doi: 10.1002/prp2.799. 34355529 PMC8343220

[53] Gilsbach R, Hein L. Are the pharmacology and physiology of α 2 adrenoceptors determined by α 2 -heteroreceptors and autoreceptors respectively? Br J Pharmacol. 2012;165(1):90–102. doi: 10.1111/j.1476-5381.2011.01533.x 21658028 PMC3252969

[54] Gilsbach R, Röser C, Beetz N, et al. Genetic dissection of α 2 -aDRENOCEPTOR FUNCTIONS IN ADRENERGIC VERSUS NONADRENERGIC CELls. Mol Pharmacol. 2009;75(5):1160–1170. doi: 10.1124/mol.109.054544 19251826

[55] Nelson LE, Lu J, Guo T, et al. The α2-adrenoceptor agonist dexmedetomidine converges on an endogenous sleep-promoting pathway to exert its sedative effects. Anesthesiology. 2003;98(2):428–436. doi: 10.1097/00000542-200302000-00024 12552203

[56] Kumar VM, Vetrivelan R, Mallick HN. Noradrenergic afferents and receptors in the medial preoptic area: neuroanatomical and neurochemical links between the regulation of sleep and body temperature. Neurochem Int. 2007;50(6):783–790. doi: 10.1016/j.neuint.2007.02.004 17403554

[57] Gazzola C, Magner T, Lisle AT, et al. Effects of α-adrenoceptor agonists and antagonists on metabolic rate in cattle. Comp Biochem Physiol A Physiol. 1995;111(1):73–77. doi: 10.1016/0300-9629(95)98522-i 7735911

[58] Miettinen TJ, Kanto JH, Salonen MA, et al. The sedative and sympatholytic effects of oral tizanidine in healthy volunteers. Anesth Analg. 1996;82(4):817–820. doi: 10.1097/00000539-199604000-00024 8615503

[59] Blaak EE, van Baak MA, Kempen KP, et al. Role of alpha- and beta-adrenoceptors in sympathetically mediated thermogenesis. Am J Physiol. 1993;264(1):E11–E17. doi: 10.1152/ajpendo.1993.264.1.E11 8094272

[60] Malanga G, Reiter RD, Garay E. Update on tizanidine for muscle spasticity and emerging indications. Expert Opin Pharmacother. 2008;9(12):2209–2215. doi: 10.1517/14656566.9.12.2209 18671474

[61] Honda M, Sekiguchi Y, Sato N, et al. Involvement of imidazoline receptors in the centrally acting muscle-relaxant effects of tizanidine. Eur J Pharmacol. 2002;445(3):187–193. doi: 10.1016/s0014-2999(02)01664-3 12079683

[62] Kino Y, Tanabe M, Honda M, et al. Involvement of supraspinal imidazoline receptors and descending monoaminergic pathways in tizanidine-induced inhibition of rat spinal reflexes. J Pharmacol Sci. 2005;99:52–60. doi: 10.1254/jphs.fp0050520 16127244

[63] Manthous CA, Hall JB, Olson D, et al. Effect of cooling on oxygen consumption in febrile critically ill patients. Am J Respir Crit Care Med. 1995;151(1):10–14. doi: 10.1164/ajrccm.151.1.7812538 7812538

